# Progress in Medicine

**Published:** 1894-12-08

**Authors:** 


### PROGRESS IN MEDICINE.

### DISEASES OF THE NERVOUS SYSTEM.

(*Continued from page* 155.)

**Amyotrophic Lateral Sclerosis.**—Senator[15] records a case which he considers supports Leyden's as against Charcot's view, Leyden maintaining that there is no possibility of distinguishing progressive muscular atrophy from amyotrophic lateral sclerosis. Senator's patient exhibited during life paralysis and contracture of the upper and lower extremities, the muscles atrophied, the tendon reflexes exaggerated, &c. No disturbance of sensibility, nor paralysis of sphincters; the electrical reactions were normal. Post-mortem examination of the spinal cord revealed atrophy of a large number of ganglionic cells in the anterior columns, especially in the cervical and upper dorsal region, but in the lumbar region the cells were normal. The anterior nerve roots were slightly atrophied. There was no trace whatever of any inflammation. The lateral columns in particular were absolutely free from sclerosis. In commenting upon the case, Leyden said that from it we are justified in concluding that contractures may exist without any lesion of the white substance, all that is needed for contracture being an interruption of continuity of the conductors which transmit to the muscles the orders of the will.

**Hæmorrhagic Necrosis of Spinal Cord.**—Van Gieson[16] calls attention to cases in which, either associated with very acute myelitis or after a traumatism, long slender columns of necrosis are discovered, if death occurs at a considerable interval of time. Two rapidly fatal cases enabled him to determine that this necrosis was consequent upon similarly situated hæmorrhages in which the blood takes the course of least resistance. The effused blood is absorbed and cavities in the cord are left behind. This condition, distinct from syringomyelia, Van Gieson has named hæmato-myeloporus.

**Unilateral Bulbar Palsy.**—A. Wiener, of New York, reports[17] an example of this rare condition, giving also an excellent description of the state of the medullary nuclei when examined post-mortem. The case occurred in a lad, seventeen years of age, in a sub-acute manner, and remained stationary for about four months until shortly before death, when dyspnœic attacks and tachycardia were added to the previously existing paralysis. Briefly the case presented the phenomena of unilateral palsy of the tongue, soft palate, pharynx and larynx on the right side, shortly before death advancing into an incomplete bilateral palsy. There was no paralysis of the lips, but great atrophy of the right half of the tongue. There was no paralysis of common sensation nor of taste. Microscopical examination of the nuclei in the medulla showed that the nuclei of the twelfth nerve on the right side were very much diseased, that on the left only slightly so; the nuclei of the eleventh (vago-accessorius) and tenth slightly affected, rather more on the right side than on the left; nucleus of the ninth on the right side only very slightly affected. The "respiratory bundle" was completely degenerated upon the right side, while on the left, in the region of the hypoglossal nucleus, only its lower and outer portions were diseased. Wiener's main conclusions from the case are: (1) The hypoglossal nucleus gives origin to fibres which supply the tongue, palate, pharynx, and larynx on one side of the body; (2) the column of fibres known as the "respiratory bundle" consists of fibres derived from the glosso-pharyngeal, vagus, and vago-accessorius nerves. (3) The glosso-pharyngeal nerves seem to control the reflexes of nausea and gagging in the soft palate and pharynx, and also to send some motor filaments to the pharyngeal muscles. These latter filaments originate in the hypoglossal nucleus and ascend in the respiratory column to the nucleus proper, making their exit with the glosso-pharyngeal nerve. (4) The soft palate muscles are not innervated by fibres from the seventh nerve.

**Efferent Fibres from the Cerebellum.**—At the late International Medical Congress Aldren Turner and Ferrier[18] described the results of experimental lesions of the cerebellum in monkeys. *Inter alia* they say they are unable to confirm Marchi's statements as to the existence of a direct efferent cerebellar tract in the antero-lateral columns of the spinal cord or of degeneration of the anterior nerve roots. In two cases of lateral lobe extirpation, however, they observed degeneration in the anterior and lateral columns of the spinal cord respectively in the position indicated by Marchi. But in the case in which there was degeneration in the anterior column, the nucleus of Deiter's on the same side was implicated, while in that in which degeneration in the lateral column was present there was a lesion of the Pons Varolii involving the nucleus of the lateral fillet. At the annual meeting of the British Medical Association at Bristol, the subject of degenerations secondary to experimental and other lesions of the cerebellum was discussed. Risien Russell[19] detailed the results of his experiments. After destruction of one lateral lobe it was found that there existed (along with other tracts of degeneration also) degenerated fibres in the inferior peduncle, which, however, could hardly be traced beyond the superior pyramidal decussation. Agreeing with Ferrier and Aldren Turner, he differed from Marchi, and could not trace fibres from this peduncle to the ascending root of the fifth nerve and spinal nerves by the descending antero-lateral tract; he also agreed with Ferrier and Turner that injury to adjacent parts was the cause of degeneration of the antero-lateral tract as observed by Marchi. Dr. Alfred W. Campbell contributed five cases of cerebellar disease. More than one of these cases showed degeneration of the direct cerebellar tract, and of the postero-external columns, a condition not before seen and not very easily explicable. Campbell confirmed Marchi's views that after hemi-lateral cerebellar lesion degenerated fibres could be traced into the roots of nearly all the cranial nerves; he saw such degeneration repeatedly.

**Pituitary Body (Functions of).**—Vassale and Sacchi[20] give a summary of their communication to the Congress at Rome. Results varied according as the gland was wholly or partly removed. They show (*a*) fall of temperature to sub-normal, permanent when excision was complete; (*b*) anorexia, listlessness, and progressive hebetude; (*c*) fibrillary twitchings and tremors of the muscles; (*d*) respiratory spasms, and (*e*) death. Many of the symptoms abated if pituitary extract was injected. The authors conclude that the pituitary produces a secretion which is of profound importance in the nutrition of the nervous and neuro-muscular systems mainly. W. L. Woollcombe[21] records a case which, upon post-mortem examination, was found to be one of psammomoma of pituitary body. A girl, aged eleven years, had had amblyopia (without optic neuritis) and vertical headache for five months. She was very depressed and apathetic, expression vacant, took no notice of or interest in anything, muscularly feeble but not paralysed, temperature subnormal, no anæsthesia, and great and progressive emaciation; later there were symptoms of pressure upon the optic commissure. The pituitary body was found to be about the size of an ordinary hen's egg. These two records—one experimental and one clinical—very closely bear out the views which Lloyd Andriezen[22] stated as to what might be expected to occur from destruction of the pituitary body.

**Tumour of Optic Thalamus.**—Masing[23] records a case occurring in a boy aged fifteen. Tremor, followed by weakness of extremities of right side, then headache, vomiting, disturbance of speech, diplopia, and right-sided convulsive seizure was the order of development of the symptoms. When seen by Masing the lad was paretic on the entire right side of the body, including the face and tongue; sensibility was also impaired in the same area, but muscle-sense was preserved, and there was no ataxia. The eyes were in a position of slight divergence. The diplopia involved the whole visual field. There was slight drooping of the left upper lid. The external ocular muscles supplied by the third nerve on either side were all paretic. The pupils equal, of moderate size, failed to react to light. There was no hemianopsia. The left cerebral peduncle was concluded to be the seat of the neoplasm. After death, however, the left optic thalamus was found to be replaced by a tumour measuring about 4 cm. long and 3 cm. broad; on microscopical examination it proved to be a sarcoma. On the floor of the aqueduct of Sylvius were two small extravasations of blood, to which the oculo-motor palsy was ascribed. The derangement of sensibility and mobility were believed to be due to transmitted pressure upon the left internal capsule and cerebral peduncle. The right cerebral peduncle was normal. Though the pulvinar was profoundly involved there was no hemianopsia.

**Optic Neuritis in Cerebral Tumour.**—Dr. J. Taylor,[5] at the Harveian Society, said it was difficult to formulate any rule as to the occurrence of optic neuritis in cases of cerebral tumour, but when the tumour was in the cerebellum, optic neuritis was almost invariably present; when it was confined to the pons or medulla, especially if it was of the infiltrating variety, it was more often absent than present; and when it was in the hemispheres, sometimes it was present and sometimes absent. A large tumour might not cause neuritis, while severe neuritis might exist with a very small growth. The rapidity of the growth of an intra-cranial tumour seemed to be an important element in the production of neuritis, and probably increased intra-cranial pressure had a close causal relation to the optic neuritis.

[15] Med. Week, March 30, 1894. [16] Med. Week, May 18, 1894, [17] New York Med. Journ., July 14, 1894. [18] Lancet, April 7, 1894. [19] Brit. Med. Journ., September 22, 1894. [20] Contralb. f. allg. Path., May, 1894, quoted in Ep. Brit. Med. Journ., June 23, 1894. [21] Brit. Med. Journ., June 23, 1894. [22] Brit. Med. Journ., Jan. 13, 1894. [23] Quoted in Amer. Journ. Med. Sc., June, 1894. [24] Lancet, April 25, 1894.

